# Comparison of continuous infusion versus bolus injection of factor concentrates for blood management after total knee arthroplasty in patients with hemophilia

**DOI:** 10.1186/s12891-017-1720-0

**Published:** 2017-08-22

**Authors:** Young Shil Park, Won-Ju Shin, Kang-Il Kim

**Affiliations:** 1 Department of Pediatrics, Kyung Hee University Hospital at Gangdong, School of Medicine, Kyung Hee University, 892, Dongnam-ro, Gangdong-Gu, Seoul, 05278 Korea; 2 Department of Orthopedic Surgery, Kyung Hee University Medical Center, 23, Kyungheedae-ro, Dongdaemun-gu, Seoul, 0244 Korea; 3 Department of Orthopedic Surgery, Kyung Hee University Hospital at Gangdong, School of Medicine, Kyung Hee University, 892, Dongnam-ro, Gangdong-Gu, Seoul, 05278 Korea

**Keywords:** Hemophilia a, Total knee arthropathy, Continuous infusion, Bolus injection, Blood

## Abstract

**Background:**

Total knee arthroplasty (TKA) has become the treatment of choice for end-stage hemophilic arthropathy of the knee. Theoretically in hemophilia A, perioperative continuous infusion (CI) of factor VIII (FVIII) would provide a more consistent FVIII level than general bolus injections (BI) in TKA. Current study was designed to evaluate the effectiveness of CI of coagulation factor concentrates during the perioperative period compared to BI.

**Methods:**

A total of 42 TKAs were performed in 31 patients with severe hemophilia A. Under the supervision of a multidisciplinary hemophilia team, CI and BI were monitored during application of a standardized regimen. Perioperative clinical parameters including postoperative hemoglobin drop, drained blood volume, transfusion rate, total consumption of FVIII, and perioperative complications were assessed.

**Results:**

The difference in the postoperative hemoglobin drop was significant between two groups with a lower decrease in the CI group (*p* = 0.002). The drained blood volume for postoperative 24 h was significantly lower in the CI than the BI groups (*p* = 0.037). Total consumption of factor concentrates for postoperative 5 days was greater in the CI group than in the BI group (*p* = 0.000). One postoperative hematoma and wound dehiscence occurred in BI group and no other complication developed.

**Conclusions:**

Although good control of hemostasis could be achieved using either method during the perioperative period of TKA, CI seems more tolerable and effective than BI to provide perioperative blood management undergoing TKA in patients with hemophilia.

**Trial registration:**

The study was retrospectively registered in WHO ICTRP under identifier KCT0002404 (date of registration: August 04, 2017).

## Background

Blood management has been an issue in perioperative care in general TKA and especially in hemophilia, inappropriate perioperative blood management in the hip and knee arthroplasty is the most important cause of affecting the clinical outcome [1–4]. Control of bleeding episodes, and the management of perioperative bleeding in patients with hemophilia, is attained via the administration of plasma-derived or recombinant coagulation factors. Most often, these factors are given at regular intervals as a bolus injection (BI) to attain hemostasis [5]; however, there has been growing interest in the use of continuous infusions (CI) of clotting factors during major surgery to maintain constant factor concentrations, thus reducing the risk of bleeding caused by overly low factor trough levels [5–8]. Compared with BI, the CI method is believed to reduce the total FVIII dose required and also the incidence of severe bleeding events. However, there has been paucity in the literature regarding comparison between these two different methods of coagulation factor replacement for major surgery in patients with hemophilia. Therefore, we questioned whether CI has superiority in terms of perioperative bleeding control and consumption of FVIII following TKA. This study was performed to explore the effectiveness and safety of CI in hemophilia A patients that underwent TKA, by comparing CI with BI.

## Methods

This prospective study involved 42 TKAs performed in 31 patients with severe hemophilia A, admitted to undergo TKA by a single institute. The inclusion criteria were severe hemophilia A (FVIII: C < 1%), more than 150 exposure days (EDs) to any FVIII product before enrolment, no previous history of inhibitors, and good compliance. Each group consisted of 21 cases. Since this disease entity is rare compared to primary osteoarthritis, we assumed that the same infusion protocol in consecutive patients would be better to manage in hemophilia teams. The BI protocol was applied to the former half of the cases in the period between July 2010 and December 2013 and the CI protocol was applied to the latter half of cases during the period between January 2014 and July 2016.

### Hemostatic treatment

Under the supervision of a multidisciplinary hemophilia team of our institute, CI followed a standardized protocol. On the day of surgery, 30 min before the incision, a bolus of FVIII concentrates was administered. The first bolus CI dose was 50 IU/kg, affording FVIII plasma levels of 100%. The infusion rate commenced at 4 IU/kg/h and was adjusted to the clinical situation by measuring FVIII levels daily. Factor concentrates were administered without additional dilution, changing the syringes every 8 h (using peripheral venous access). CI was performed for 5 days. During the first 3 days after the operation, FVIII infusion rates were controlled to achieve an FVIII level of 100%. After the first 3 days of operation, the infusion rate was decreased to achieve target FVIII levels of 80% and tailored based on the patient status. In some cases with lower FVIII levels relative to target factor levels, a BI was prescribed in parallel. BI was also administered using the standard protocol of our center; the first bolus of 50 IU/kg was given 30 min before surgery to achieve a preoperative FVIII level of 100%; BI continued for 3 days after operation [9]. Approximately 50% of the initial dose was administered every 8 h. The doses were changed depending on the patient’s postoperative status, and the dosing intervals were increased to 12 h after postoperative 7 days. From postoperative 6 days, all patients were applied to BI protocol for easier rehabilitation.

The total consumption of coagulation factor concentrates during hospitalization was recorded for each procedure. An elastic compression stocking was applied to prevent deep vein thrombosis. No other chemical antithrombotic prophylaxis was prescribed.

### Procedures

All operations were performed by a single surgeon (corresponding author) under the general anesthesia with use of a tourniquet. Conventional cemented TKA prostheses were used in all cases. Postoperative drainage was constant and maintained for 2 days. Postoperative rehabilitation began as soon as patients returned to their rooms, using a passive mobilization device. Identical peri- and post-operative care was delivered to both groups. All patients had same postoperative rehabilitation and recommended to discharge at postoperative 10 days.

### Evaluation of outcomes and complications

The level of hemoglobin was evaluated the day before TKA, on the operation day, and on postoperative days 1, 2, and 5. To evaluate postoperative blood loss, the volume of drained blood was assessed from patient records. Data collected on short-term complications included hemarthrosis, acute infection, and CI-related complications (such as inhibitor development and thrombotic event) till the postoperative 6 months.

### Statistical analysis

All eligible patients were enrolled and their data used to evaluate efficacy and safety. Student’s t-test and Fisher’s exact test (the chi-squared test) were employed for between-group comparisons. A *P* value <0.05 was considered statistically significant.

## Results

### Patient characteristics

In total, 42 cases in 31 patients with severe hemophilia A were evaluated. The mean age of patients at the time of operation was 39.4 ± 9.332 years (range: 26.0–64.0 years). Eleven patients had bilateral TKAs, while one of these patients underwent TKA under BI once and under CI at another time. The first consecutive 21 patients underwent TKA under BI and the latter 21 patients under CI. Recombinant FVIII concentrates were infused to three patients in the CI group, and plasma-derived FVIII concentrates were administered in other cases.

### Comparison between BI and CI groups

The mean age of patients at the time of operation in each group was 43.33 ± 9.593 years (range: 27.0–64.0 years) in the CI group and 35.47 ± 7.646 years (range: 26.0–62.0 years) in the BI group. Comparison of the patient characteristics of the two groups revealed no difference in underlying viral status or operation direction. The clinical characteristics of both groups are listed in Table 1.

**Table 1:** Patient characteristics of the continuous infusion group and bolus injection group

	Continuous infusion (*n* = 21)	Bolus injection (*n* = 21)	*p*
Age (years)			
Mean ± SD	43.33 ± 9.593	35.47 ± 7.646	0.006
BMI			
Mean ± SD	21.67 ± 3.028	23.67 ± 3.201	0.044
Hemoglobin (g/dL)			
Mean ± SD	14.71 ± 0.864	15.05 ± 1.963	0.468
Patient’s viral status			
HBV+	1	1	1.000
HCV+	13	13	1.000
TKA (n)			
Right	13	11	
Left	8	10	0.111

*SD* Standard deviation, *BMI* Body mass index, *HBV+* Hepatitis virus B, *HCV* Hepatitis virus B, *TKA* Total knee arthroplasty

*p < 0.05

A surgery-related decrease in hemoglobin was observed in both groups. The average preoperative hemoglobin and hemoglobin on the operation day did not differ between the groups, but comparison of the difference between the two values (hemoglobin drop) in both groups revealed a significant difference (1.14 ± 0.961 g/dL in the CI group vs. 2.26 ± 1.238 g/dL in the BI group; *p* = 0.002). The volume of drained blood during the first 24 h postoperatively differ significantly between the two groups (*p* = 0.037) (Table 2). One patient in the CI group, and five patients in the BI group, required transfusion after TKA during the perioperative period. Total consumption of factor concentrates from operation day to postoperative day 5 was 604.03 ± 112.86 IU/kg in the CI group and 467.85 ± 105.157 IU/kg in the BI group (*p* = 0.000).

**Table 2:** Comparison between the continuous infusion group and bolus injection group

	Continuous infusion (*n* = 21)	Bolus injection (*n* = 21)	*p*
Total amount of coagulation factor concentrates (IU/kg)			
Mean ± SD			
Operation day 0–3	460.23 ± 72.721	359.19 ± 86.686	0.000*
Operation day 0–5	604.03 ± 112.836	467.85 ± 101.157	0.000*
Hemoglobin (g/dL)			
Mean ± SD			
Pre-operation	14.71 ± 0.864	15.05 ± 1.963	0.468
Operation day (post)	13.66 ± 1.188	12.79 ± 1.891	0.081
Hemoglobin drop	1.14 ± 0.961	2.26 ± 1.238	0.002*
Drained blood volume at postoperation day 1 (mL)	574.76 ± 286.855	788.33 ± 351.494	0.037*
Mean ± SD			
Packed RBC transfusion			
No. (n)	1 / 21	5 / 21	0.184
Volume (mL/kg)	0.81 ± 3.740	3.56 ± 7.015	0.120

*SD* Standard deviation, *RBC* Red blood cell

*p < 0.05

### Complications and safety

During hospitalization, one patient in the BI group had a postoperative hematoma that required aspiration. Another patient, in the BI group, presented with wound dehiscence which healed after re-closure. No acute periprosthetic joint infection or other thrombotic events were observed in either group. There was no thrombotic event despite the absence of antithrombotic prophylaxis. In addition, no new inhibitor development occurred in either the BI or CI group during the postoperative 6 months.

## Discussion

Postoperative hemorrhagic complications in hemophilia patients are quite common and may lead to serious consequences after TKA [10–13]. In patients with hemophilia, coagulation factor concentrates can be administered by way of CI or BI as the perioperative blood management [14]. While several studies have shown that the CI method is safe and effective due to maintaining constant level of the coagulation factor in the blood during the perioperative periods [15–17], most studies practically have utilized a simple BI method [2, 12, 18]. Furthermore, no comparative study regarding CI and BI methods following total joint replacement for of hemophilia could be found. Therefore the authors attempted to evaluate whether the CI could show efficacy and advantages compared to the BI especially undergoing a relatively common major surgery, such as TKA by way of prospective comparative study. We confirmed that CI could lead to a significant decrease in both postoperative hemoglobin drop and drained blood volume following TKA.

CI has been established that coagulation factor concentrates are stable and microbiologically safe at room temperature [19, 20]. Moreover, it has been known that Cl is a cost-effective drug delivery approach, compared with BI [6, 21]. In this study, the average consumption of coagulation factor concentrates with CI for 5 days was 604.03 ± 112.84 IU/kg, in comparison with 467.85 ± 1.16 IU/kg of BI for the same period after undergoing TKA. There was a discrepancy in our results vis-à-vis the theoretically-advantageous aspect of CI. The discrepancy seems to be coming from the dissimilar BI protocols implemented by different institutions. In this study, the target peak level of factor concentrates administered via BI was 100% at Day 0–3 and 80% at Day 4–6. This regimen required a less amount of factor concentrates than other regimens as reported from various centers [22–26]. Unlike CI that maintains the peak level for 24 h a day, BI achieves the peak level only for a certain duration of just injected time. Afterward, the FVIII levels, given by BI, continue to decline for the half-life of FVIII, 8–12 h, until FVIII level rises after administration of a dose to reach the peak level again. The fact of the matter is that the peak level using a BI method is maintained not 100%, but just 75% for 3 days, in comparison with the conception applicable to CI method. Therefore, the total factor dose administered by BI might be lesser than that of CI, which would maintain the peak level 100% throughout the 24 h-duration. However, the presumption is that the inability to maintain the peak level for 100% of the time would lead to the inferior results of BI, as shown by the decline in hemoglobin level or blood drainage volume, as well as by the increased frequency of blood infusion. Furthermore, a less dose of factor concentrates was administered via BI in this same regimen, in comparison with hemophilia patients who had undergone total hip arthroplasty [9]. This would be the rationale behind CI showing greater consumption of FVIII than BI in this investigation. Further study on various protocols, including cost-effectiveness, is necessary.

The initial concern for prolonged CI was focused on factor stability and safety when the products were kept at room temperature for some period of time [27]. However, such a concern has been addressed in several articles [19, 20, 23]. The potential for inhibitor development has been a recent issue in the context of CI; as such development renders FVIII replacement therapy ineffective and makes it difficult to control bleeding [14, 28, 29]. At the end of our study, we found that no patient had developed inhibitors of FVIII. However, at the time of enrollment, all subjects had over 150 EDs FVIII with no history of prior development of inhibitors were taken. Other studies also reported that inhibitors were absent in previously treated patients who underwent CI during surgery [22].

Another issue with CI is a thromboembolism. Recent reviews on CI found that venous thrombosis developed in 1.39%–3.8% of patients who had received FVIII via CI during various surgeries [16, 30]. In this study, no symptomatic thrombotic event was noted, although we did not use chemoprophylaxis. Also, neither saline dilution nor heparin addition was not carried out during the CI. The suggestion is that CI would not increase the risk of postoperative thrombosis. No saline dilution was prepared for CI in this study, while the syringe was changed at 8 h intervals. CI was carried out at the infusion rate of 4 IU/kg/h of clotting factor concentrates.

In current study, the patients in the BI group showed a significant drop in hemoglobin levels after TKA. Moreover, there was a significant difference between the CI and BI groups in drained blood volume for postoperative 24 h. The frequency number of postoperative transfusions given by BI was larger than that provided by CI. Meanwhile, the patient age and body mass index before surgery were not similar between these two groups. During the investigational period, the study was conducted in sequence of patient’s hospital admission. The difference of the mean ages of these two groups might inadvertently contribute to bias. It appears to be an issue, attributing to the scarcity of the disease and that of patients. How this may affect the bleeding management of patients with hemophilia remains unclear, but the difference between the two groups was the limitation in this study. Another limitation of this study was the fact that it had not been a randomized study. However, it was prospectively designed and followed up. Finally, this study was carried out among patients in Asian population and there could be ethnic differences. Further study regarding Western population would be interesting.

## Conclusions

Good control of hemostasis could be achieved using either infusion method during the perioperative period of TKA in patients with hemophilia. However, CI seems more tolerable and effective than BI to reduce perioperative blood loss in these cohorts undergoing TKA.

## Abbreviations

BI: bolus injections
CI: continuous infusion
EDs: exposure days
FVIII: factor VIII
TKA: Total knee arthroplasty
